# ‘Spotted Nanoflowers’: Gold-seeded Zinc Oxide Nanohybrid for Selective Bio-capture

**DOI:** 10.1038/srep12231

**Published:** 2015-07-16

**Authors:** Veeradasan Perumal, U. Hashim, Subash C.B. Gopinath, R. Haarindraprasad, K.L. Foo, S.R. Balakrishnan, P. Poopalan

**Affiliations:** 1 Biomedical Nano Diagnostics Research Group, Institute of Nano Electronic Engineering (INEE), University Malaysia Perlis (UniMAP), Kangar, Perlis, Malaysia; 2 School of Microelectronic Engineering, University Malaysia Perlis (UniMAP), Kuala Perlis, Perlis, Malaysia

## Abstract

Hybrid gold nanostructures seeded into nanotextured zinc oxide (ZnO) nanoflowers (NFs) were created for novel biosensing applications. The selected ‘spotted NFs’ had a 30-nm-thick gold nanoparticle (AuNP) layer, chosen from a range of AuNP thicknesses, sputtered onto the surface. The generated nanohybrids, characterized by morphological, physical and structural analyses, were uniformly AuNP-seeded onto the ZnO NFs with an average length of 2–3 μm. Selective capture of molecular probes onto the seeded AuNPs was evidence for the specific interaction with DNA from pathogenic Leptospirosis-causing strains via hybridization and mis-match analyses. The attained detection limit was 100 fM as determined via impedance spectroscopy. High levels of stability, reproducibility and regeneration of the sensor were obtained. Selective DNA immobilization and hybridization were confirmed by nitrogen and phosphorus peaks in an X-ray photoelectron spectroscopy analysis. The created nanostructure hybrids illuminate the mechanism of generating multiple-target, high-performance detection on a single NF platform, which opens a new avenue for array-based medical diagnostics.

The creation of new nanostructures enabling nanoscale chemical/biological interactions has opened a wide range of applications. These applications currently play a vital role in interdisciplinary sciences^1^,^2^,^3^,^4^. In addition, nanotextured surfaces have been proposed for nanohybridization using a defined surface covered with nanostructures^5^. Nanoscale structures combined with noble metals are expected to yield novel materials that will create new avenues for diagnosis and therapeutics. These nano-creations, with different sensing properties including optical, electronic, and magnetic abilities, can be used as part of a semiconductor-based sensing system. Furthermore, metal nanoparticles have been used for high-performance sensing through the production of hybrid nanocrystals^6^,^7^. These novel nanostructures coupled with metal nanoparticles are expected to enhance optical and electronic properties^8^,^9^. Noble metal nanostructures possess unique properties, such as high sensitivity, chemical stability and bio-compatibility^10^,^11^. Computational modelling and experimental evidence have indicated that the doping density influences the sensitivity of bio-recognition^12^,^13^. Thus, nanohybrids are a new functional class and represent one of the most promising candidates for the exploration of new applications^14^. Zinc oxide (ZnO) has received much attention over the past decade due to its unique optical and electronic properties as an important metal oxide semiconductor derived from the group II–VI series in the periodic table^15^. ZnO has a wide bandgap of 3.37 eV and a large exciton binding energy of 60 meV^16^. Studies that focused on the morphological aspects of ZnO nanostructures have found these materials to be useful because of their ease of synthesis, high crystallinity with low structural defects and low processing temperatures. ZnO also possesses excellent electrical characteristics, suitable for the development of fast and accurate sensors^17^,^18^,^19^. The biocompatibility characteristics of ZnO make this material a convenient choice for conducting surface functionalization and interfacing with chemical and biological compounds at pH extremes^20^. Nanohybrids that comprise ZnO and noble metal nanoclusters have attracted tremendous interest in recent years owing to their potential for improved catalytic activity, excellent surface-to-volume ratio and several functionalities that are superior to those of pure ZnO nanomaterials^20^,^21^. However, gold nanoparticles (AuNPs) have high electron affinity, and the Schottky barrier produced between AuNPs and various metals is high^22^. Because of the unique characteristics of gold (Au), such as suitable with surface chemical functionalization, biocompatibility, anti-oxidative characteristics, high conductivity and the ability to tailor Au to uniform and different nano-sizes, this metal has been actively researched^23^,^24^,^25^,^26^. Therefore, the agglutination of AuNPs into a ZnO nanostructure enables thiolated biomolecules to bind easily, directly and selectively. These compatibilities permit the selective binding of a nucleic acid-based probe to the agglutinated AuNP for the specific bio-recognition of DNA hybrids expressed in the bacterial genome of pathogenic strains of *Leptospira interrogans* which leads to leptospirosis. Leptospirosis is an epidemic bacterial disease that is caused by spirochetes (specifically, *Leptospira* species), which infect humans and other species of vertebrates. Pathogenic *Leptospira* is the prime causative agent of severe pulmonary haemorrhage, acute respiratory failure, myocarditis, meningitis and renal failure in humans^27^,^28^. Furthermore, given the existence of more than three hundred *Leptospira* serovars, distinguishing between pathogenic and non-pathogenic strains is mandatorily required^29^. Currently, no published studies have been conducted on *Leptospira* detection using nanostructured biosensors. To address these issues, a specific gene that is expressed only in pathogenic *Leptospira*: Hemolysis-associated protein-1 (Hap1)^30^—was selected for probe design and interactive analyses were performed by hybridization and mis-matching. This detection strategy was implemented on a metal-oxide-nanoparticle-agglutinized nanowire, creating ‘spotted nanoflowers’ (NFs) that provide a sensing platform for multiple applications.

## Results and Discussions

**Figure 1:**
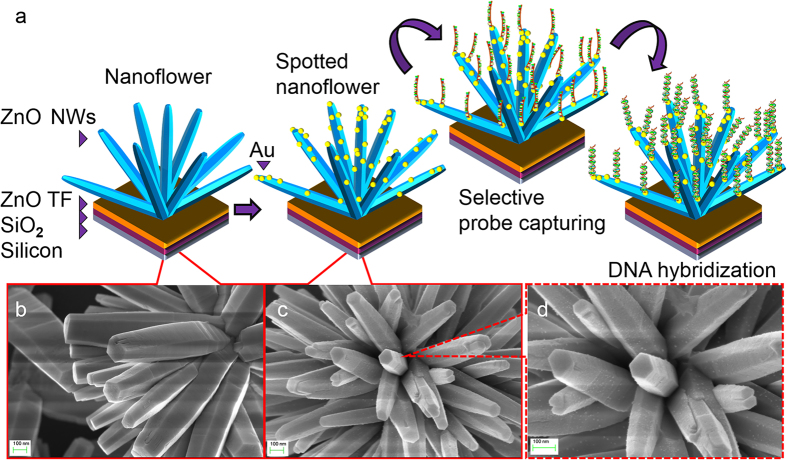
(**a**) Schematic illustration of the steps involved in the synthesis of the spotted NF DNA bioelectrode. (**b**) FESEM image of low magnification revealing the flower-like ZnO nanostructure possessing hexagonally shaped tips, which demonstrates the high crystallinity of the prepared ZnO nanowire ends. (**c**,**d**) Low- and high-magnification images of spotted NFs indicate that radially oriented NFs have an average length of 2–3 μm and a diameter of approximately 100 nm.

To investigate the effect on the conduction mechanism of agglutinized AuNP layers of various thicknesses, AC impedance spectroscopic analyses were performed. The results depicted in Supplementary Fig. 1a show that the impedance and dielectric constant decreased as the thickness of the AuNP seeding increased (0, 10, 20 and 30 nm), whereas the conductivity varied in the opposite manner and increased (Supplementary Fig. 1b). These variations were attributed to the grain sizes and dipole dynamics^31^. However, a contrasting trend was observed with the 40-nm-thick AuNP layer. Supplementary Fig. 2 presents the characteristic photoluminescence manifested by ZnO NFs before and after Au sputtering. In the bare ZnO NFs, a weak near-band-edge UV emission at 379 nm and a broad, strong green emission peak at 550 nm were observed. Upon Au sputtering, the UV emission intensity drastically increased whereas the green emission was significantly reduced. Moreover, the near-band-edge UV emission intensity increased with the increase in the thickness of the AuNPs. As shown in the inset (Supplementary Fig. 2), the enhancement factor of the UV emission reaches a maximum value with a 30-nm sputter thickness and then decreases with a further increase to a AuNP thickness of 40 nm. A reason for this attenuation is that the increase in the thickness of the AuNP layer causes the accumulation of AuNPs on the surface of the ZnO NFs, eventually forming a continuous film. Thus, the adsorption of AuNPs leads to a non-radiative dissipation of surface plasmon which suppresses photon emission^32^. Thus, the agglutination of 30 nm of AuNP was selected for this study from the different thicknesses of AuNPs analysed. The bifunctional ZnO-Au nanohybrids offer unprecedented nanostructures, high catalytic activity, high sensitivity and stability for use in biosensing applications. A systematic study on the energetics of ZnO-Au nanohybrid systems is important to tailor the properties of next-generation bio-nano devices. Hence, in this study we characterized the intrinsic properties of ZnO-Au nanohybrids morphologically and structurally. The selective bio-capture of thiolated probe DNA and hybridization with complementary target DNA is illustrated in Fig. 1a. To further elucidate the biosensing capacity of these novel nanohybrids, we examined their biomolecular interactions using impedance spectroscopy.

### Morphological features of ZnO-Au nanohybrids

#### Field emission scanning electron microscopy (FESEM)

The surface morphology of the Au-doped ZnO nanostructure was characterized via FESEM. The FESEM images depicting the morphological features of ZnO nanostructures obtained before and after Au sputtering are presented in Fig. 1b,c, respectively. Figure 1c,d shows the complete structure of the nanowire, which forms a flower-like structure with Au seeded in this nanostructure, causing it to have the appearance of a ‘spotted flower’ (henceforth referred to as a ‘spotted NF’). Clear agglomerations of the AuNPs were observed on all the petals of the spotted NF, demonstrating their homogeneity. The spotted NF morphologies of the ZnO matrix were substantially coarsened due to the attachment of AuNPs and this attachment contributed to an increased surface area. It is expected that the reduction in defects caused by oxygen vacancies is possible due to this significant increase in surface area. This improvement facilitates the chemisorption of organic molecules on the substrate and will accommodate high numbers of DNA molecules during the immobilization and hybridization processes^33^.

#### Transmission electron microscopy (TEM)

**Figure 2:**
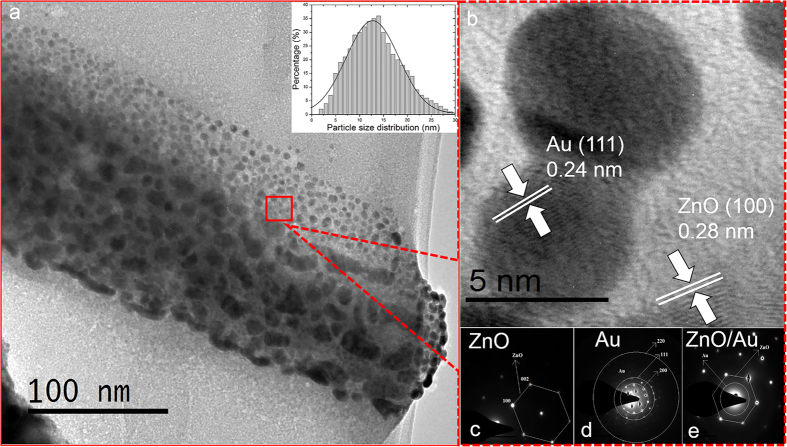
(**a**) Typical TEM micrograph of spotted NFs. The inset graph is a histogram with normal distribution showing the particle size distribution (**b**) A high-resolution TEM image showing the lattice fringes of Au and ZnO on spotted NF (**c**) Selected area electron diffraction pattern of the ZnO nanowire illustrated a hexagonal spot pattern corresponding to (100) and (002) planes, (**d**) the crystallography plane of AuNPs indexed to (111), (200) and (220) and (**e**) SAED pattern of Au and ZnO NFs that indicate the hybrid structure of the spotted NF.

The structural morphology of the spotted NF composites was further confirmed by TEM. Figure 2 reveals TEM images at both low and high magnifications and selected area electron diffraction pattern (SAED) of the fabricated spotted NF. The magnified TEM images in Supplementary Fig. 3 indicate that the average length of the spotted NF is 2–3 μm, and the diameter is approximately 100 nm. The AuNPs, which were relatively well deposited onto the highly crystalline ZnO NF surface, are seen in the low-magnification TEM image (Fig. 2a) as dark, contrasting spherical, dumbbell and irregularly shaped structures. The AuNPs have a mean diameter of 10.6 nm, with a 2.8 to 28.7 nm min-max at a sputtered thickness of 30 nm, as seen in the inset of Fig. 2a. The high-resolution TEM (HRTEM) image of the region shown in Fig. 2a is illustrated in Fig. 2b. The image also reveals that a typical AuNP deposited on the NFs has a diameter of approximately 10 nm. This image also demonstrates the lattice fringes of the spotted NFs owing to the interplanar lattice spacing of 0.24 nm and 0.28 nm corresponding to the Au (111) crystal planes and ZnO (100) planes, respectively^34^. This information suggests that the AuNPs exist in a face-centred cubic structure. Figure 2c shows the corresponding SAED pattern of ZnO, which indicates the presence of a highly crystalline ZnO wurtzite structure, whereas Fig. 2d shows a different orientation of crystallography planes denoting the polycrystalline nature of the deposited AuNPs^35^,^36^. The corresponding SAED patterns of Au and ZnO NFs indicate the hybrid structure of the spotted NF as shown in Fig. 2e.

### Structural characterization

#### X-ray diffraction (XRD)

**Figure 3:**
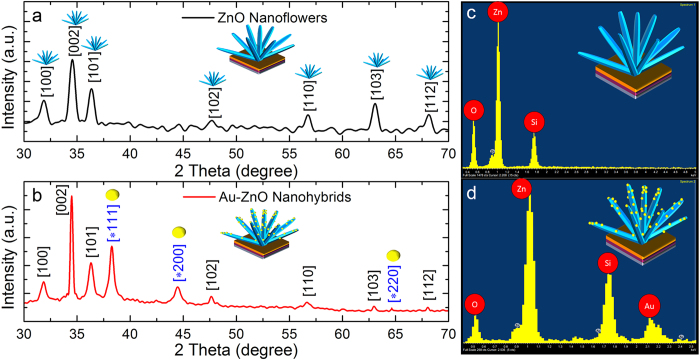
(**a**,**b**) X-ray diffraction and (**c**,**d**) energy-dispersive X-ray spectra of ZnO NF before and after Au sputtering.

An XRD analysis was carried out to study the crystal quality, size, plane orientation and morphology of the fabricated spotted NFs. As shown in Fig. 3a,b, the diffractograms of spotted NFs were compared with those of pure ZnO NFs. The diffraction peaks corresponding to Au and ZnO were matched with the reference spectra of ZnO JCPDS Card No. 36-1451 and bulk Au JCPDS Card No. 65-2879. The obtained XRD patterns have reflection peaks at 31.86° (100), 34.49° (002), 36.34° (101), 47.63° (102), 56.65° (110), 62.96° (103) and 68.04° (112) (Fig. 3a) and thus can be classified as the wurtzite phase of the ZnO nanostructure. Thus, the resultant planes demonstrated that the pure hexagonal wurtzite ZnO structures were synthesized with high-quality crystals and c-axis alignment. Figure 3b shows the XRD spectra of the spotted NFs with three additional diffraction peaks compared to the pure wurtzite ZnO nanocrystals (Fig. 3a). The diffraction peaks at 38.27°, 44.49° and 64.6° are assigned to diffraction lines of (111), (200) and (220), respectively, which index as the face-centred cubic of Au^37^,^38^. The results were in good agreement with the results obtained from SAED and HRTEM. The crystallite structures of ZnO and AuNPs were estimated using the Scherrer equation. The crystallite size of the ZnO nanowire was 54.7 nm whereas the AuNPs were 9.6 nm, in good agreement with the HRTEM results.

#### Energy-dispersive X-ray spectroscopy (EDX)

The EDX spectrum of the fabricated ZnO-NF-Au nanohybrids is presented in Fig. 3c,d. The EDX spectrum demonstrates the presence of oxygen (O) and zinc (Zn) peaks in the ZnO NFs, as shown in Fig. 3c. There is a strong Au peak present together with the Zn and O peak in the spotted NF, as shown in Fig. 3d. These results confirm the deposition of AuNPs on hydrothermal-grown spotted NFs. Furthermore, this result demonstrates the absence of other impurities in the prepared sample.

### Selective bio-capture

**Figure 4:**
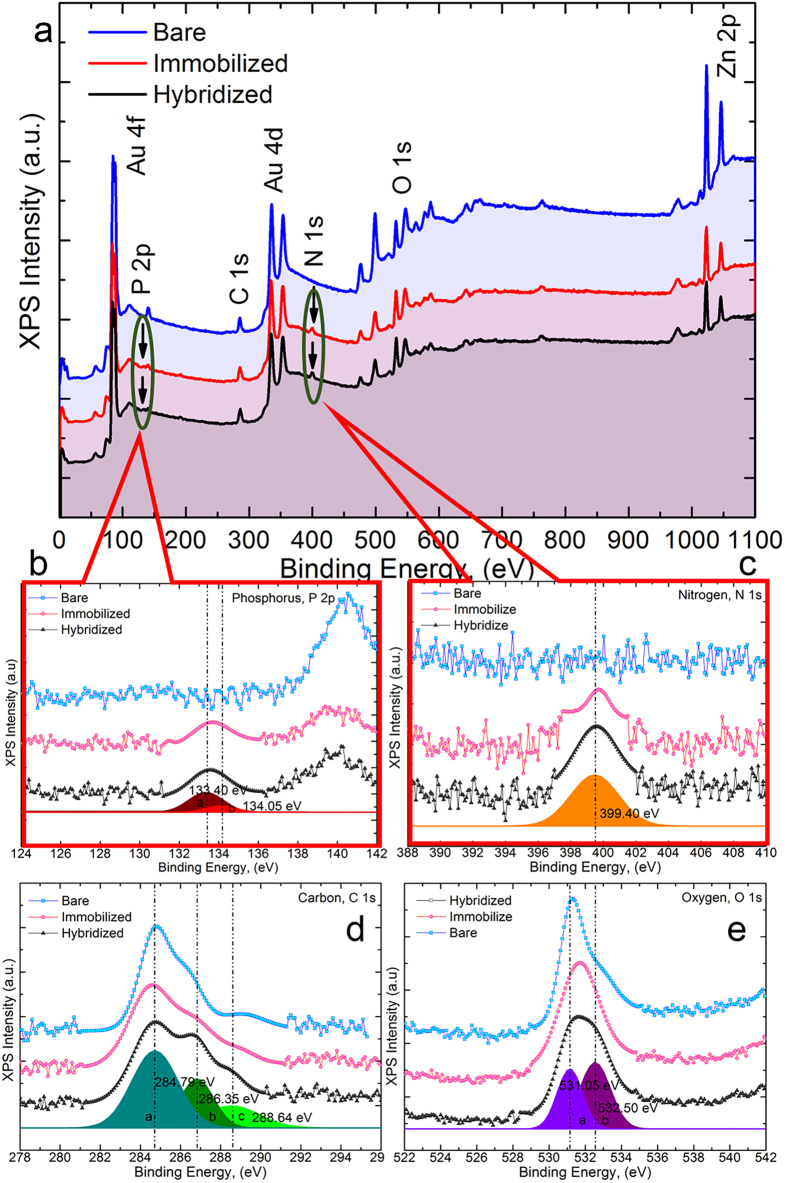
(**a**) Survey scan of XPS core level spectra taken on a spotted NF, immobilized (spotted NF/p-DNA) and hybridized (spotted NF/p-DNA/t-DNA) bioelectrode. (**b**) Binding energy of P, P2p (**c**) N, N1s (**d**) C, C1s and (**e**) O, O1s electrons.

X-ray photoelectron spectroscopy (XPS) studies were performed to investigate the elemental composition present on the outermost layer of the spotted NFs and its degree of transformation on the surface upon the immobilization and hybridization of DNA designed for the detection of *Leptospira*. These interactions were analysed by XPS, and the core level XPS spectra of spotted NFs, spotted NF/p-DNA (probe) and spotted NF/p-DNA/t-DNA (duplexed) bioelectrodes are shown in Fig. 4. In Fig. 4a, a wide XPS survey scan revealed the presence of carbon (C), O, Zn, and Au in all the samples analysed. The Au, Au4f and Zn, Zn2p peaks were recorded at higher resolution from spotted NFs, as shown in Supplementary Fig. 4. The Zn2p indicates binding energies of 1022.6 eV and 1045.8 eV, corresponding to a Zn^2+^ valance state. Binding energies of 83.6 eV and 87.8 eV corresponding to Au4f_7/2_ and 4f_5/2_, respectively, were also observed. The variation in the Au4f_7/2_ binding energy compared to the 84.0 eV of bulk Au demonstrated the strong electronic interaction between the AuNPs and ZnO NFs. Phosphorus (P) and nitrogen (N) photoelectron peaks also existed for the spotted NF/p-DNA and spotted NF/p-DNA/t-DNA samples. The XPS spectra in the present study have photoelectron peaks of Zn, O and Au from the spotted NF, whereas peaks observed for elements C, N, P correspond to the functional groups of the DNA molecule, which consists of a phosphodiester deoxyribose backbone and bases (adenine, thymine, cytosine and guanine)^39^,^40^,^41^,^42^. These results confirm the presence of DNA molecules in the spotted NF/p-DNA and spotted NF/p-DNA/t-DNA samples. As shown in Fig. 4(a–e), the intensity of each element decreases as the immobilization and hybridization process occurs, mainly due to the higher inelastic mean free path (IMFP) attributed to the layer of DNA molecules adhering on the sample surface^43^,^44^. This substantiates the idea that the DNA molecules are successfully immobilized and hybridized on the spotted NF bioelectrode. Although the deconvoluted P2p profile shown in Fig. 4b has a lower signal-to-noise ratio, it is possible to detect the peak upon immobilization and hybridization. P2p can be interpreted as a strong confirmation of the bond formation between the DNA oligomer and Au substrate^42^,^43^. The N1s spectrum is shown in Fig. 4c, where a clear difference in the photoelectron peak formation upon immobilization and hybridization can be seen. In addition, there is also a shift of binding energy between the immobilization and hybridization results. This shift is ascribed to the bond formation between ssDNA and dsDNA on the surface of the spotted NF^40^,^41^. The core level binding energies of O1s and C1s, which resolved into several peaks, are shown in Fig. 4d,e. These elements, along with binding energy and assignment, are shown in Supplementary Table 1. Hence, these XPS results suggested that DNA molecules were successfully immobilized and hybridized on the spotted NF surface.

### Biomolecular interaction analyses by impedance spectroscopy

**Figure 5:**
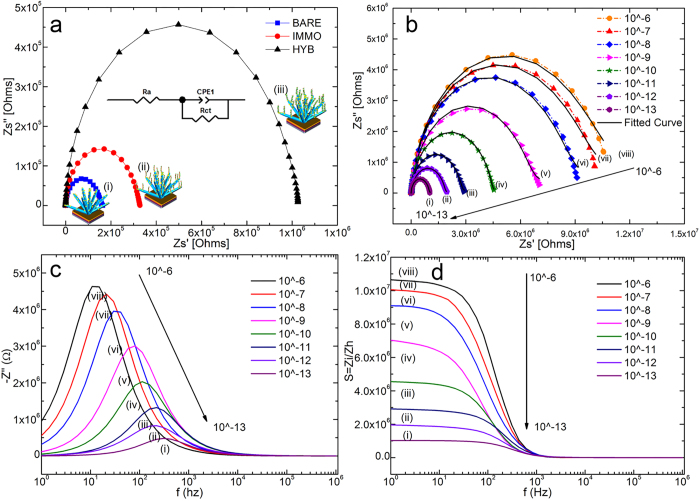
(**a**) Impedance spectra of (i) spotted NF, (ii) spotted NF/p-DNA (probe) and (iii) spotted NF/p-DNA/t-DNA (duplex) bioelectrode, the inset shows the Randles equivalent circuit, where the parameters Ra, Rct and CPE represent the bulk solution resistance, charge transfer resistance and constant phase element, respectively (**b**) Impedimetric response curve of spotted NF/p-DNA hybridized with different concentrations of complementary target DNA (i-viii) 10 μM to 100 fm, (**c**) Imaginary part showing the overall impedance, which decreases, and the peak frequency, which is shifted towards the higher frequencies as the concentration of complementary DNA decreases. (**d**) The gain curve of spotted NF/p-DNA hybridized at different concentrations.

Nyquist plots of the AC impedance spectra for the samples of spotted NFs, spotted NF/p-DNA and spotted NF/p-DNA/t-DNA are shown in Fig. 5a. The obtained Nyquist plot can be expressed with a Randles equivalent circuit (Fig. 5a inset), where the parameters Ra and Rct represent the bulk solution resistance and charge transfer resistance respectively while CPE is an abbreviation for constant phase element. The spotted NF morphology of a ZnO matrix which is substantially coarsened due to the attachment of AuNPs has contributed to high surface roughness at the interfacial layer of the sample. Such changes in the electrode surface may cause nonhomogeneity which subsequently leads to variability in the relaxation times. Hence, double-layer capacitance CPE were chosen instead of pure capacitance. The semicircle (i) in the impedance spectrum represents the interfacial charge transfer resistance, Rct (~0.16 MΩ), corresponding to the carrier transfer from the modified electrode to the ferricyanide in the solution (Fig. 5a). The results suggest that the interfacial layer of bare a spotted NF has an excellent electron transfer ability towards the electrolyte solution because of its high surface area. An increase in the Rct (~0.35 MΩ) value with respect to the bare spotted NF was observed upon the immobilization (semicircle (ii)) of SH-ssDNA probe. Thus, the observed increase in diameter is explained due to the adsorption of the probe DNA immobilized by the Au-SH bond on the surface, thus blocking the diffusion of [Fe(CN)_6_]^3–/4–^ to the electrode surface. The observation of such an increase not only confirms the effective immobilization of the probe DNA but also reveals the electrostatic repulsion caused by the electro-negative phosphate skeleton of the DNA. Furthermore, a corresponding increase in the Rct (1.10 MΩ) value for the spotted NF/p-DNA/t-DNA was observed (semicircle (iii)). This is caused by an increment in the negatively charged phosphate skeleton of DNA resulting from the hybridization process, leading to an increase in electrostatic repulsive force between the negatively charged phosphate backbone and the [Fe(CN)_6_)]^3–/4–^ anions at the electrode surface. Therefore, the resultant Rct value confirms the successful hybridization of the target DNA on the probe-immobilized bioelectrode. Figure 5b depicts the Nyquist plot of the probe DNA-modified bioelectrode exposed to various concentrations of target DNA (10^−6^ to 10^−13^ M). As seen in Fig. 5b, the Rct value increased upon hybridization with increasing concentrations of target DNA. The increase in the DNA concentration leads to an increase in the repulsion between the redox anion and the phosphate backbone of the probe molecule which appears as a significant increase in the charge transfer resistance, Rct. Supplementary Table 2 displays the simulation results with nonlinear curve fitting for the measured impedance with an equivalent circuit. Figure 5c depicts the imaginary part of the impedance by plotting ‘Z’ against the logarithm of frequencies which exhibit the relaxation frequencies. The decrease in the imaginary part of the impedance indicates that the conductivity of the sensor increases, whereas the shift indicates that the DNA concentration decreases and the relaxation time increases. Thus, decreasing the DNA concentration results in an overall decrease in the imaginary part of the impedance, which reflects the ease of flow for the charge carriers within the AC electric field^31^,^45^. Additionally, the effects of both the complementary DNA concentration and frequency on the sensitivity of the spotted NF bioelectrode are shown in Fig. 5d. The sensing mechanism of a spotted NF can be described starting from the fact that oxygen molecules from the ambient atmosphere can be easily adsorbed onto the ZnO NFs owing to the large surface area–to-volume ratio of the ZnO nanostructures. This leads to a migration of electrons from the conduction band of ZnO towards oxygen, creating a depletion layer with lower conductivity (O_2_ (gas) + e^−^ = O_2−_ (ads))^38^,^45^. Therefore, the ZnO nanostructure agglutinized with AuNP is a system in which the Fermi energy level of ZnO is lower than that of Au. This energy-level disparity is a consequence of the fact that the work function of ZnO is higher (5.2–5.3 eV) than that of Au (5.1 eV), resulting in an electron transfer from Au to ZnO until the two systems reach a dynamic equilibrium^36^,^37^. The electron transfer leads to an ohmic junction at the agglutination point due to a decrease in the ZnO depletion layer with a higher conductivity. Conversely, the selective capture of the immobilized thiolated probe DNA onto the AuNP alters the dynamic equilibrium obtained after the Au agglutination. The covalent bond between Au and sulphur^46^ gives rise to a hole current density on the ZnO surface induced by the negatively charged phosphate backbone. This results in a decrease in the electron concentration again leading to broadening of the depletion layer, which lowers the conductivity.

### Analytical performance of a spotted NF biosensor

**Figure 6:**
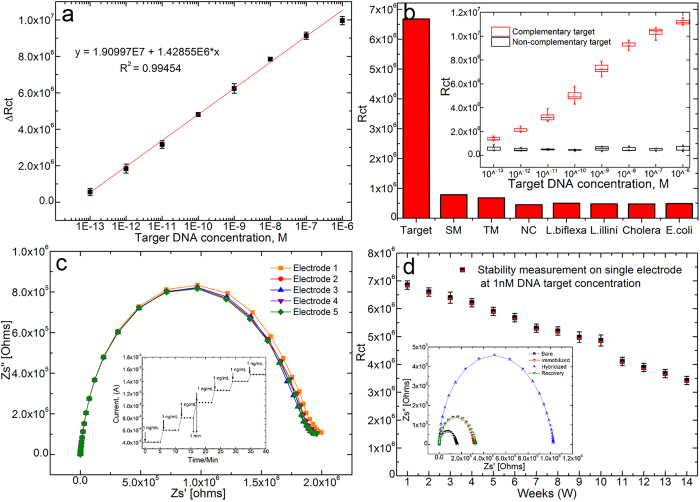
(**a**) Shows the linear regression curve for different concentrations of target DNA with linear equation ∆Rct = 1.456E6x + 1.915E7, (R^2^ = 0.995). (**b**) The bar chart shows the specificity of the spotted NF bioelectrode towards mismatching and cross hybrids; the inset shows a box plot of complementary and non-complementary target oligonucleotides hybridized to the immobilized probe oligonucleotides at different target oligonucleotide concentrations (**c**) The reproducibility curve is shown with 5 parallel bioelectrodes prepared under similar processing conditions; the inset shows the electroanalytical curve for the 1 min response time. (**d**) Showing the stability of the spotted NF biosensor. Inset shows the regeneration Nyquist plot for the spotted NF biosensor.

We further tested the analytical performance of spotted NF bioelectrodes, as shown in Fig. 6(a–d). In brief, the spotted NF bioelectrode has good linearity, and a detection limit of 100 fM was estimated using a signal-to-noise ratio of more than 3σ^47^,^48^. The Rct value of the t-DNA (1 nM) was ∼6.7 MΩ, which was nearly 8.5 times larger than that of single-base-mismatched DNA (∼0.78 MΩ) at the same concentration, indicating that the DNA biosensor has excellent sequence specificity towards even a single base mismatch. Therefore, the specificity reported is among the best result reported compared to previous literatures^48^,^49^,^50^. The cross-specificity with other bacteria and non-pathogenic *Leptospira* species/serovar is also shown in Fig. 6b. The value of the Rct signal does not vary significantly in the presence of unrelated molecules indicating non-influence of the individual interferants. The reproducibility curve (Fig. 6c) of the developed spotted NF bioelectrode has a relative standard deviation (RSD) of 3% with 5 parallel measurements prepared under similar processing conditions. The electro-analytical response of the spotted NF bioelectrode is shown in the inset of Fig. 6c, revealing that the response time for this DNA sensor can be 1 min with complete duplex formation^51^. The stability results (Fig. 6d) indicated that the prepared bioelectrode is very stable and loses only 70% of its activity, even after 10 weeks. As shown in the inset, the Rct values for the bioelectrode both before and after regeneration were found to be the same, with negligible differences. After 10 regenerations and hybridizations, the electrode lost only approximately 9.3% of its original Rct signal value. Therefore, the regeneration of the proposed DNA sensor possesses potential for repeated monitoring of target DNA.

## Conclusions

In the field of nanotechnology, nanostructures, nanohybrids, nanoelectronics, nanotextures and nanosensors are important candidates in bottom-up and top-down approaches to lead the way towards interdisciplinary sciences. In this study, we combined these disciplines and created a novel spotted flowered nanowire for impedance sensing to distinguish pathogenic and non-pathogenic *Leptospira* species. A clear demonstration of femtomolar sensitivity was obtained. Furthermore, stability, reproducibility and regeneration of this sensing surface were demonstrated. These appealing characteristics of distributed AuNPs on ZnO NFs enable the immobilization of different probes for human pathogen detection and can act as a single platform for multiple detection strategies. This approach could be potentially expanded to an array-based technology for high-throughput and multiple diagnoses.

## Methods

### Materials and reagents

ZnO seed solution sol gel was prepared using zinc acetate dihydrate [Zn(CH_3_COO)_2_.2H_2_O] (98%; Sigma Aldrich, St. Louis, Missouri, USA) in ethanol solvent (EtOH; 99.99%; J.T. Baker, Center Valley, Pennsylvania, USA). Monoethanolamine (MEA; 99%; Merck, Kenilworth, New Jersey, USA) was used as a stabilizer. Hydrochloric acid (HCl; 37%; J.T. Baker, Center Valley, Pennsylvania, USA), aqueous ammonia (NH_4_OH; 30%; J.T. Baker, Center Valley, Pennsylvania, USA), hydrogen peroxide (H_2_O_2_; 30%; J.T. Baker, Center Valley, Pennsylvania, USA) and DI water were used to prepare standard cleaning 1 (RCA1) and standard cleaning 2 (RCA2) solutions. Buffered oxide etchant (BOE; 6:1; J.T. Baker, Center Valley, Pennsylvania, USA), negative photoresist (NR7-6000PY; Futurrex, Franklin, New Jersey, USA) and resist developer (RD6; Futurrex, Franklin, New Jersey, USA) were used for the photolithography. The growth solution was prepared by mixing equal concentrations (25 mM) of zinc nitrate hexahydrate (99%; Sigma Aldrich, St. Louis, Missouri, USA) and hexamethylenetetramine (99%; Merck, Kenilworth, New Jersey, USA) in DI water. A 0.05 M phosphate-buffered saline (PBS) solution was prepared from 0.1 M sodium phosphate buffer (Sigma Aldrich, St. Louis, Missouri, USA) with 0.15 M sodium chloride (NaCl, Sigma Aldrich, St. Louis, Missouri, USA) and the pH was adjusted to 7.4. Sterile double-distilled and deionized water from Sigma Aldrich was used throughout the experiments. Tris-EDTA buffered solution (pH 7.4, Sigma Aldrich, St. Louis, Missouri, USA) was used to dilute the oligonucleotides. Finally, sodium dodecyl sulphate (SDS; 98%; Sigma Aldrich, St. Louis, Missouri, USA) was used for the removal of unbound probe or target. Potassium ferricyanide [K_3_Fe(CN)_6_] and potassium ferrocyanide [K_4_Fe(CN)_6_] (Sigma Aldrich, St. Louis, Missouri, USA) were used for the impedance measurements. All other chemicals were of analytical grade and used without further purification. The oligonucleotides were purchased from First BASE laboratories Sdn. Bhd (Selangor, Malaysia). The 21-mers oligonucleotide sequences used in the present work were as follows:

Thiolated probe DNA (p-DNA) - 5’-SH-(CH_2_)_3_-CCG TGA TTT TCC TAA CTA AGG -3’; complementary target DNA (t-DNA):5’-CCT TAG TTA GGA AAA TCA CGG -3’; non-complementary target DNA (nc-DNA):5’-GAC CTT TGA TTT TCA TTC TTA -3’; one-base mismatching target DNA (m-DNA):5’- CCT TAG TTA GGA ATA TCA CGG -3’; three-base mismatching target DNA (tm-DNA): 5’- CCT TAG TTA GGA TTT TCA CGG -3’;

The single-strand thiolated probe DNA (p-DNA) was designed from HAP1. The HAP1 nucleotide gene sequence used to design the probe in this study was obtained from GenBank database. The whole genomic sequence can be found in GenBank (http://www.ncbi.nlm.nih.gov/nuccore/AF366366.1).

### Fabrication of interdigitated electrodes (IDEs)

A p-type silicon wafer was cleaned using RCA1, RCA2 and BOE to remove organic and inorganic contaminants and the native oxide layer on the wafer surface^52^. Next, the silicon wafer was rinsed and cleaned with deionized water. An SiO_2_ layer approximately 200 nm thick was produced on the cleaned wafer surface using a wet oxidation furnace. Using a conventional lithography process, an IDE device of 7 mm × 5 mm in size was patterned using negative resists (NR7-6000PY) on the SiO_2_/Si substrate. A thermal evaporator (Auto 306 thermal evaporator; Edwards High Vacuum International, Wilmington, MA, USA) was used to deposit a titanium/Au (500/3000 Å) layer on the SiO_2_/Si substrate and the layer was patterned using a lift-off process. Eventually, the negative photoresist sacrificial layer which formed was removed using acetone. In this work, an IDE with 16 fingers was fabricated, in which the width and length of each finger were 0.1 and 3.9 mm, respectively, and the spacing between the two adjacent fingers was 0.1 mm.

### Preparation of ZnO NFs

ZnO NFs (ZnO NFs) were prepared as follows. First, 8.78 g of Zn(CH_3_COO)_2_.2H_2_O was dissolved in 200 ml of ethanol solvent (ZnO seed solution sol gel). The concentration of ZnO was kept constant at 0.2 M. The mixed solution was then vigorously stirred with a magnetic stirrer at 60 °C for 30 min. The MEA stabilizer was added drop by drop to the ZnO solution with constant stirring for 2 h. Finally, the transparent and homogenous solution was stored for aging at room temperature for 1 day. The aged ZnO sol gel was deposited onto the IDE device drop by drop using a spin coating technique at a speed of 3000 rpm for 20 s. The deposition process of the seed layer was repeated 3 times to obtain a thicker ZnO thin film (ZnO TFs). For each deposition process, the coated ZnO TFs were dried at 150 °C for 20 min to remove the organic residuals that might exist on the ZnO thin films. The coated ZnO TFs were then annealed in a furnace under ambient air at 300 °C for 2 h to obtain highly crystallized ZnO. For the hydrothermal growth of ZnO NFs, the prepared substrate with the coated seed layer was submerged backward inside the growth solution using a Teflon sample holder. Equal concentration (25 mM) growth solution was prepared by mixing both zinc nitrate hexahydrate and hexamethyltetramine in deionized water. The growth process was completed inside an oven at 93 °C for 5 h. The prepared hydrothermally grown ZnO NFs were cleaned with isopropanol and deionized water to remove residual salts prior to annealing in a furnace under ambient air at 300 °C for 2 h.

### Preparation of ZnO-NF-Au nanohybrids

ZnO-NF-Au nanohybrids were prepared using a sputtering method. To form the ZnO-NF-Au nanohybrids, 10, 20, 30 and 40 nm Au wetting layers were physically deposited by a Sputter coater (EMS550X) with a Au target and a rotating stage. The detailed experimental conditions were as follows: electric current was maintained at 25 mA for 2–8 min with vacuum pressure of Argon process level at 10^−2^ mbar. This process allowed us to obtain Au-decorated ZnO NFs forming ZnO-NF-Au nanohybrids.

### Pathogenic *Leptospira* DNA immobilization and hybridization

The ZnO-NF-Au nanohybrid electrodes were cleaned and dried using nitrogen and used to fabricate DNA biosensors to detect *Leptospira*. The direct immobilization of probe DNA onto the electrode surface was achieved by dispensing 10 μL of 1 μM probe DNA solution in Tris-EDTA buffer for 3 h followed by washing the electrode and then rinsing with sterile double-distilled water. The DNA hybridization was accomplished by dispensing the probe modified electrodes into different concentrations of target DNAs (complementary, non-complementary and mismatches). The electrodes were washed after the hybridization process to remove any unbound target before any measurements were taken. The complete immobilization process of thiolated probe DNA and hybridization with target molecules is schematically illustrated in Fig. 1. The electrodes’ hybridized DNA was regenerated by rinsing the surfaces with hot (95 °C) deionized water for 2 min, followed by rapid cooling in an ice bath. The surface was repetitively hybridized and regenerated with target molecules for reusability testing. The stability of the probe DNA electrodes was studied for 4 weeks by performing the assay on a daily basis. Electrodes were stored at 4 °C when not in use.

### Microscopic nano imaging

The morphology and structural properties of the ZnO-NF-Au nanohybrid samples were investigated using FESEM (Carl Zeiss AG ULTRA55, Gemini). Further nanoscale imaging was performed with the aid of TEM. High-resolution TEM (HRTEM) images and SAED of the ZnO-NF-Au nanohybrids were acquired using a Philips CM-200 Twin with incident energy of 200 keV.

### Structural analysis

XRD (Bruker D8, Bruker AXS, Inc., Madison, WI, USA) with a Cu Kα radiation (λ = 1.54 Ǻ) was used to study the crystallization and structural properties of ZnO-NF-Au nanohybrids. XRD patterns were recorded in the range of 30° to 70° operating at a voltage of 40 kV and a current of 40 mA. The XRD peak analysis was carried out using the DIFFRAC^plus^ (2003 version of the Eva 9.0 rev.0 software. The material composition, immobilization and hybridization were analysed using XPS (Omicron Dar400, Omicron, Germany). The chamber pressure was maintained at 2.4 e^−10^ Torr throughout the measurement. The obtained peaks were deconvoluted using CasaXPS software.

### Impedance spectroscopy and optical measurements

Electrical measurements of current to voltage (I–V) were taken using (Kiethley 6487 Picoammeter) and impedance spectroscopy measurements were performed with a Novocontrol alpha high-frequency analyser (Hundsangen, Germany). To characterize the ZnO-NF-Au nanohybrids, the prepared sample was immersed in PBS buffer (pH 7.4) containing a mixture of 2 mM K_3_[Fe(CN)_6_]/K_4_[Fe(CN)_6_]. The impedance spectra of the real and imaginary parts of impedance, Zs’ and Zs”, were obtained by sweeping the frequency of 1–100 MHz with an applied AC amplitude of 1 V RMS. All the measurements were recorded at room temperature. The sensitivity of the fabricated spotted NF sensor was evaluated as a function of frequency and complementary DNA concentrations according to the following relationship in equation 1:


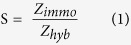


Where Z_immo_ represents the real part of impedance upon immobilization probe DNA and Z_hyb_ represents the real part of impedance after hybridization of complementary DNA under different concentrations. The impedimetric DNA biosensor sensitivity is evaluated from the slope of the linear plot between the value of ∆Rct and the concentration of t-DNA.

In addition, the optical and luminescence properties of the ZnO-NF-Au nanohybrids were studied through photoluminescence fluorimetry (PL, Horiba Fluorolog-3, HORIBA Jobin Yvon Inc., USA). The PL spectra of sample were recorded at different angle and position to assure the result is not influence by sample non-homogeneity.

## Additional Information

**How to cite this article**: Perumal, V. *et al.* 'Spotted Nanoflowers': Gold-seeded Zinc Oxide Nanohybrid for Selective Bio-capture. *Sci. Rep.* **5**, 12231; doi: 10.1038/srep12231 (2015).

## Supplementary Material

Supplementary Information

## References

[b1] Simpson R. E. *et al.* Interfacial phase-change memory. Nat. Nanotech. 6, 501–505 (2011). 10.1038/nnano.2011.96 21725305

[b2] Kolobov A. V., Krbal M., Fons P., Tominaga J. & Uruga T. Distortion-triggered loss of long-range order in solids with bonding energy hierarchy. Nat. Chem. 3, 311–316 (2011). 21430691 10.1038/nchem.1007

[b3] Nomura K. *et al.* An angular fluidic channel for prism-free surface-plasmon-assisted fluorescence capturing. Nat. Commun. 4, 2855 (2013). 24335751 10.1038/ncomms3855

[b4] Bang D. *et al.* Mirror-symmetric Magneto-optical Kerr Rotation using Visible Light in [(GeTe)2(Sb2Te3)1]n Topological Superlattices. Sci. Rep. 4, 1–7 (2014). 10.1038/srep05727 PMC4101470 25030304

[b5] Gopinath S. C. B., Awazu K., Tominaga J. & Kumar P. K. R. Monitoring biomolecular interactions on a digital versatile disk: A BioDVD platform technology. ACS Nano 2, 1885–1895 (2008). 19206429 10.1021/nn800285p

[b6] Dong A., Chen J., Vora P. M., Kikkawa J. M. & Murray C. B. Binary nanocrystal superlattice membranes self-assembled at the liquid-air interface. Nature 466, 474–477 (2010). 20651688 10.1038/nature09188

[b7] Liu M. *et al.* An anisotropic hydrogel with electrostatic repulsion between cofacially aligned nanosheets. Nature 517, 68–72 (2015). 25557713 10.1038/nature14060

[b8] Chang. W. *et al.* Hard gap in epitaxial semiconductor–superconductor nanowires. Nat. Nanotech. (2015), http://dx.doi.org/10.1038/nnano.2014.306. 10.1038/nnano.2014.306 25581886

[b9] Li K., Liu X., Wang Q., Zhao S. & Mi Z. Ultralow-threshold electrically injected AlGaN nanowire ultraviolet lasers on Si operating at low temperature. Nat. Nanotech. 10, 140–144 (2015). 10.1038/nnano.2014.308 25599190

[b10] Jiang R., Li B., Fang C. & Wang J. Metal/semiconductor hybrid nanostructures for plasmon-enhanced applications. Adv. Mater. 26, 5274–5309 (2014). 24753398 10.1002/adma.201400203

[b11] Solanki P. R., Kaushik A., Agrawal V. V. & Malhotra B. D. Nanostructured metal oxide-based biosensors. NPG Asia Mater. 3, 17–24 (2011).

[b12] Li J., Zhang Y., To S., You L. & Sun Y. Effect of Nanowire Number, Diameter, and Doping Density on Nano-FET Biosensor Sensitivity. ACS Nano 5, 6661–6668 (2011). 21815637 10.1021/nn202182p

[b13] Nair P. R. & Alam M. A. Design Considerations of Silicon Nanowire Biosensors. IEEE T. Electron Dev 54, 3400–3408 (2007).

[b14] Song Z., Chang H., Zhu W., Xu C. & Feng X. Rhodium Nanoparticle-mesoporous Silicon Nanowire Nanohybrids for Hydrogen Peroxide Detection with High. Sci. Rep. 5, 7792 (2015). 25588953 10.1038/srep07792 PMC4295103

[b15] Kashif M. *et al.* Morphological, optical, and Raman characteristics of ZnO nanoflakes prepared via a sol-gel method. Phys. Status Solidi 209, 143–147 (2012).

[b16] Baruah S. & Dutta J. Hydrothermal growth of ZnO nanostructures. Sci. Technol. Adv. Mater. 10, 013001 (2009). 10.1088/1468-6996/10/1/013001 PMC5109597 27877250

[b17] Foo K. L., Kashif M., Hashim U. & Liu W.-W. Effect of different solvents on the structural and optical properties of zinc oxide thin films for optoelectronic applications. Ceram. Int. 40, 753–761 (2014).

[b18] Foo K. L., Hashim U., Muhammad K. & Voon C. H. Sol – gel synthesized zinc oxide nanorods and their structural and optical investigation for optoelectronic application. Nanoscale Res. Lett. 9, 1–10 (2014). 25221458 10.1186/1556-276X-9-429 PMC4150024

[b19] Tak M., Gupta V. & Tomar M. Flower-like ZnO nanostructure based electrochemical DNA biosensor for bacterial meningitis detection. Biosens. Bioelectron. 59, 200–7 (2014). 24727606 10.1016/j.bios.2014.03.036

[b20] Geng J., Song G. H., Jia X. D., Cheng F. F. & Zhu J. J. Fast one-step synthesis of biocompatible ZnO/Au nanocomposites with hollow doughnut-like and other controlled morphologies. J. Phys. Chem. C 116, 4517–4525 (2012).

[b21] Lee J., Shim H. S., Lee M., Song J. K. & Lee D. Size-Controlled Electron Transfer and Photocatalytic Activity of ZnO À Au Nanoparticle Composites. J. Phys. Chem. Lett. 2, 2840–2845 (2011).

[b22] Pawinrat P., Mekasuwandumrong O. & Panpranot J. Synthesis of Au–ZnO and Pt–ZnO nanocomposites by one-step flame spray pyrolysis and its application for photocatalytic degradation of dyes. Catal. Commun. 10, 1380–1385 (2009).

[b23] Lim Z.-Z. J., Li J.-E. J., Ng C.-T., Yung L.-Y. L. & Bay B.-H. Gold nanoparticles in cancer therapy. Acta Pharmacol. Sin. 32, 983–990 (2011). 21743485 10.1038/aps.2011.82 PMC4002534

[b24] Upadhyayula V. K. K. Functionalized gold nanoparticle supported sensory mechanisms applied in detection of chemical and biological threat agents: A review. Anal. Chim. Acta 715, 1–18 (2012). 22244163 10.1016/j.aca.2011.12.008

[b25] Guirgis B. S. S. *et al.* Gold nanoparticle-based fluorescence immunoassay for malaria antigen detection. Anal. Bioanal. Chem. 402, 1019–1027 (2012). 22089818 10.1007/s00216-011-5489-y

[b26] Ryu S.-W. *et al.* Gold nanoparticle embedded silicon nanowire biosensor for applications of label-free DNA detection. Biosens. Bioelectron. 25, 2182–5 (2010). 20227871 10.1016/j.bios.2010.02.010

[b27] Branger C. *et al.* Protection against Leptospira interrogans Sensu Lato Challenge by DNA Immunization with the Gene Encoding Hemolysin-Associated Protein 1 Protection against Leptospira interrogans Sensu Lato Challenge by DNA Immunization with the Gene Encoding Protein 1. Infect. Immun. 73, 4062–4069 (2005). 15972494 10.1128/IAI.73.7.4062-4069.2005 PMC1168576

[b28] Fearnley C. *et al.* The development of a real-time PCR to detect pathogenic Leptospira species in kidney tissue. Res. Vet. Sci. 85, 8–16 (2008). 17961617 10.1016/j.rvsc.2007.09.005

[b29] Lee S. H. *et al.* Identification and partial characterization of a novel hemolysin from Leptospira interrogans serovar lai. Gene 254, 19–28 (2000). 10974532 10.1016/s0378-1119(00)00293-6

[b30] Branger C. *et al.* Identification of the hemolysis-associated protein 1 as a cross-protective immunogen of Leptospira interrogans by adenovirus-mediated vaccination. Infect. Immun. 69, 6831–8 (2001). 11598056 10.1128/IAI.69.11.6831-6838.2001 PMC100061

[b31] Saif A. A. & Poopalan P. Correlation between the chemical composition and the conduction mechanism of barium strontium titanate thin films. J. Alloy Compd. 509, 7210–7215 (2011).

[b32] Cheng C. W. *et al.* Surface plasmon enhanced band edge luminescence of ZnO nanorods by capping Au nanoparticles. Appl. Phys. Lett. 96, 3–5 (2010).

[b33] Wang X., Wang W. & Liu Y.-L. Enhanced acetone sensing performance of Au nanoparticles functionalized flower-like ZnO. Sens Actuat. B Chem. 168, 39–45 (2012).

[b34] Ahmad M. *et al.* Synthesis of hierarchical flower-like ZnO nanostructures and their functionalization by Au nanoparticles for improved photocatalytic and high performance Li-ion battery anodes. J. Mater. Chem. 21, 7723 (2011).

[b35] Wu J.-J. & Tseng C.-H. Photocatalytic properties of nc-Au/ZnO nanorod composites. Appl. Catal. B Environ. 66, 51–57 (2006).

[b36] Wang X., Kong X., Yu Y. & Zhang H. Synthesis and characterization of water-soluble and bifunctional ZnO-Au nanocomposites. J. Phys. Chem. C 111, 3836–3841 (2007).

[b37] Shan G., Zhong M., Wang S., Li Y. & Liu Y. The synthesis and optical properties of the heterostructured ZnO/Au nanocomposites. J. Colloid Interf Sci. 326, 392–5 (2008). 10.1016/j.jcis.2008.06.027 18639886

[b38] Gogurla N., Sinha A. K., Santra S., Manna S. & Ray S. K. Multifunctional Au-ZnO plasmonic nanostructures for enhanced UV photodetector and room temperature NO sensing devices. Sci. Rep. 4, 6483 (2014). 25255700 10.1038/srep06483 PMC4175732

[b39] Petrovykh D. Y., Kimura-Suda H., Whitman L. J. & Tarlov M. J. Quantitative analysis and characterization of DNA immobilized on gold. J. Am. Chem. Soc. 125, 5219–5226 (2003). 12708875 10.1021/ja029450c

[b40] Rosenberg R. A. *et al.* The relationship between interfacial bonding and radiation damage in adsorbed DNA †. Phys. Chem. Chem. Phys. 16, 15319–15325 (2014). 24943511 10.1039/c4cp01649a

[b41] Kummer K. *et al.* Real-time study of the modification of the peptide bond by atomic calcium. J. Phys. Chem. B 115, 2401–2407 (2011). 21338139 10.1021/jp109555j

[b42] Vilar M. R. *et al.* Interaction of self-assembled monolayers of DNA with electrons: HREELS and XPS studies. J. Phys. Chem. B 112, 6957–6964 (2008). 18489141 10.1021/jp8008207

[b43] Singh S. *et al.* DNA hybridization on silicon nanowires. Thin Solid Films 519, 1151–1155 (2010).

[b44] Wustoni S., Hideshima S., Kuroiwa S. & Nakanishi T. Sensitive electrical detection of human prion proteins using field effect transistor biosensor with dual-ligand binding amplification. Biosens. Bioelectron. 1–7 (2014), 10.1016/j.bios.2014.08.028. 25175745

[b45] Kashif M., Ali M. E., Ali S. M. U., Hashim U. & Hamid S. B. A. Impact of hydrogen concentrations on the impedance spectroscopic behavior of Pd-sensitized ZnO nanorods. Nanoscale Res. Lett. 8, 68 (2013). 23399029 10.1186/1556-276X-8-68 PMC3599888

[b46] Häkkinen H. The gold–sulfur interface at the nanoscale. Nat. Chem. 4, 443–455 (2012). 22614378 10.1038/nchem.1352

[b47] Ramulu T. S. *et al.* Nanowires array modified electrode for enhanced electrochemical detection of nucleic acid. Biosens. Bioelectron. 40, 258–64 (2013). 22883747 10.1016/j.bios.2012.07.034

[b48] Wang L. *et al.* Development of an electrochemical DNA biosensor with the DNA immobilization based on *in situ* generation of dithiocarbamate ligands. Bioelectrochemistry 88, 30–35 (2012). 22763422 10.1016/j.bioelechem.2012.04.003

[b49] Huang H., Bai W., Dong C., Guo R. & Liu Z. An ultrasensitive electrochemical DNA biosensor based on graphene / Au nanorod / polythionine for human papillomavirus DNA detection. Biosens. Bioelectron. 68, 442–446 (2015). 25618376 10.1016/j.bios.2015.01.039

[b50] Amouzadeh Tabrizi M. & Shamsipur M. A label-free electrochemical DNA biosensor based on covalent immobilization of salmonella DNA sequences on the nanoporous glassy carbon electrode. Biosens. Bioelectron. 69, 100–105 (2015). 25710894 10.1016/j.bios.2015.02.024

[b51] Balakrishnan S. R. *et al.* Development of highly sensitive polysilicon nanogap with APTES/GOx based lab-on-chip biosensor to determine low levels of salivary glucose. Sens Actuat. A Phys. 220, 101–111 (2014).

[b52] Adam T. & Hashim U. Highly sensitive silicon nanowire biosensor with novel liquid gate control for detection of speci fi c single-stranded DNA molecules. Biosens. Bioelectron. 67, 656–661 (2014). 25453738 10.1016/j.bios.2014.10.005

