# Supplementary material for: ‘Spotted Nanoflowers’: Gold-seeded Zinc Oxide Nanohybrid for Selective Bio-capture
Source: Sci Rep. 2015 Jul 16;5:12231. doi: 10.1038/srep12231 (PMC4503952; doi:10.1038/srep12231)
Supplement: Supplementary Information [file srep12231-s1.pdf]

Supplementary Information

'Spotted Nanoflowers': - Gold-seeded Zinc Oxide Nanohybrid for Selective Bio-capture

Veeradasan Perumal[1], U. Hashim[1,2,*], Subash C.B. Gopinath[1], R. Haarindraprasad[1], K.L.Foo[1], S.R. Balakrishnan[1] and P. Poopalan[2]

[1]Biomedical Nano Diagnostics Research Group, Institute of Nano Electronic Engineering (INEE), Universiti Malaysia Perlis (UniMAP), Kangar, Perlis, Malaysia.

[2]School of Microelectronic Engineering, University Malaysia Perlis (UniMAP), Kuala Perlis, Perlis, Malaysia.

* uda@unimap.edu.my

Analytical performances of spotted nanoflower biosensor

Sensitivity

The sensitivity of the proposed spotted nanoflower bioelecrode was investigated and a linear correlation of the differences in the charge transfer resistance were plot using equation (1):

$$\Delta \mathrm{Rct} = \mathrm{Rcthyb} - \mathrm{Rctimmo} \qquad \text{(S1)}$$

with respect to the logarithm of complementary DNA concentration are shown in Fig. 6a. It was observed that the ΔRct linearly increases with increasing of complementary DNA concentration from 10 μM-100 fM and thereafter saturated further. The difference value (ΔRct) between Rct at probe immobilized biolectrode and hybridized bioelectrode were found to be well proportional to the natural logarithm of t-DNA concentration with a linear equation of ΔRct = 1.456E6x + 1.915E7, (R^2 = 0.99542). A detection limit of detection as 100 fM was estimated using signal to noise ratio of more than 3σ (where σ is the standard deviation of the blank solution, n=5). The detection limit is much lower than previously reported gold nanowire and ZnO nanowire using electrochemical impedance spectroscopy[18,45].

Mis-matching and Specificity analyses

A high specificity is a necessity for a new designed biosensor with potential applications in complicated samples. In order to investigate the specificity of proposed biosensor, the Rct signal responses induced by different DNA strands including non-complementary, single-base mismatch DNAs and three-base mismatches DNA were measured and the results are shown in the Fig. 6b. The Rct value of the t-DNA (1nM) was ~6.7 MΩ, which was nearly 8.5 times larger than that of single base mismatched DNA (~0.78 MΩ) with the same concentration, indicating that DNA biosensor has excellent sequence specificity towards even a single base mismatched. In comparison to previously done articles by researchers, this particular result obtained is noted as the highest and thus, demonstrates the best specificity[48-50]. Similarly, with the triple mis-matches, there is a no complementation between the DNAs. The cross specificity with other bacteria and non-pathogenic Leptospira species/serovar is also demonstrated in Fig. 6b. The figure inset shows the value of Rct signal in the presence of non-complementary target compared with that of target DNA at same concentration. It was observed that the value of Rct signal does not vary significantly in the presence of unrelated molecules indicating non-influence of the individual interferants.

Reproducibility and Response time

In the present work, the reproducibility of the proposed spotted nanoflower biosensor was also investigated by comparing five samples of the same batch and from different preparations. Fig. 6c is depicting the Rct error bar value of five samples prepared under similar processing conditions. The relative standard deviation (R.S.D) of 3% with 5 parallel measurements for 1 nM complementary DNA was calculated, and it revealed a good reproducibility of the fabricated DNA biosensor.

The electroanalytical response of spotted nanoflower biosensor shown in figure inset describes the real-time detection of proposed DNA sensor with different complementary DNA concentrations. For each injection of 1 nM of DNA target, five measurements were taken continuously for 5 min to elucidate the continuity of the DNA sensor. Upon injection of complementary DNA, the current responses increases rapidly and reached 93-97% of the steady state current within 1 min, indicates the level saturation. This result suggests that the response time for this DNA sensor can be 1 min with the complete duplex formation. Supplementary 5 shows the electroanalytical response for 100 fM concentration.

Stability and Regeneration

The stability of the proposed spotted nanoflower biosensor was examined by the shelf-life study over a period of 14 weeks at a regular interval of 1 week and stored at 4°C when not in use; the results are displayed in Fig. 6d. The bioelectrode was used to detect 1 nM of t-DNA concentration at room temperature. The stability results show that prepared bioelectrode is very stable and only loss 10% of its activity (Rct value) after 4 weeks. It was observed that the prepared bioelectrode retain more 70% of its activity even after 10 weeks and subsequently loss 50 % of its activity after 14 weeks. Regeneration was accomplished by rinsing the DNA hybridized bioelectrode surfaces with hot double distilled H_2O for 2 min, and subsequently rapid cooling in ice bath. The reusability of the biosensor was tested by repetitive hybridization with t-DNA, the Rct values of the regenerated biolectrode before and after hybridization of t-DNA were obtained (Fig. 6d; inset). As shown in the inset, the Rct values for both the bioelectrode before and after regeneration were found to be same with negligible differences. The observed results suggest that thermal denaturation treatment can effectively break the hydrogen bond between the hybridized strands without desorbing the covalent Au thiol

bond from DNA immobilization on the bioelectrode surface, indicates the stability of sensing surfaces under higher temperature. After consequent 10 regenerations and hybridizations, the electrode only loss about 9.3% of its original Rct signal value. Therefore, the regeneration of the proposed DNA sensor possessed potential for repeatable monitoring of target DNA.

Supplementary Figure

Supplementary Figure 1

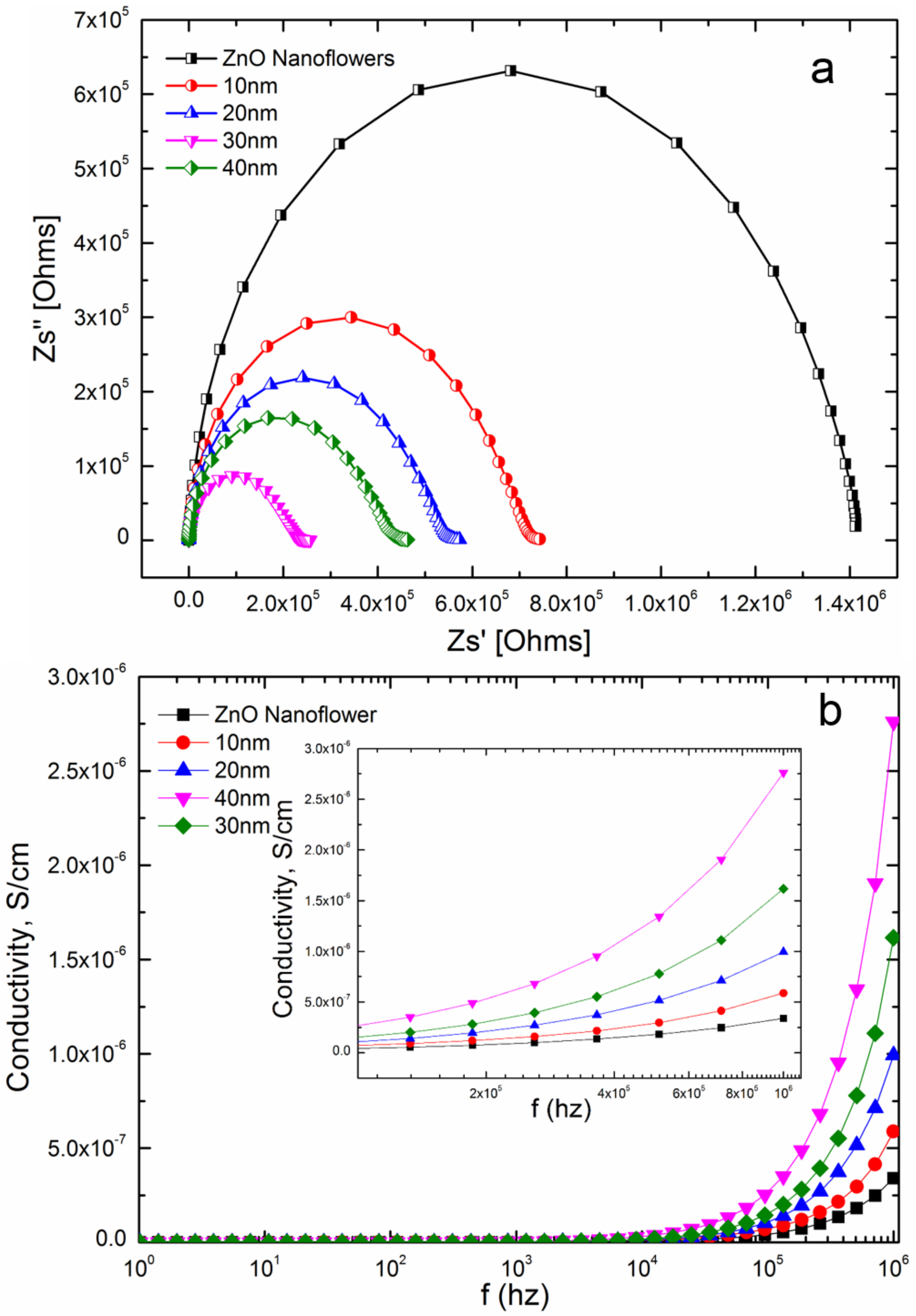

Supplementary Figure 1: The Nyquist plot of ZnO nanoflowers before and after Au sputtering with (i-iv) 10, 20, 30 and 40nm thickness respectively, b) Shows the conductivity for each sample respectively.

Supplementary Figure 2

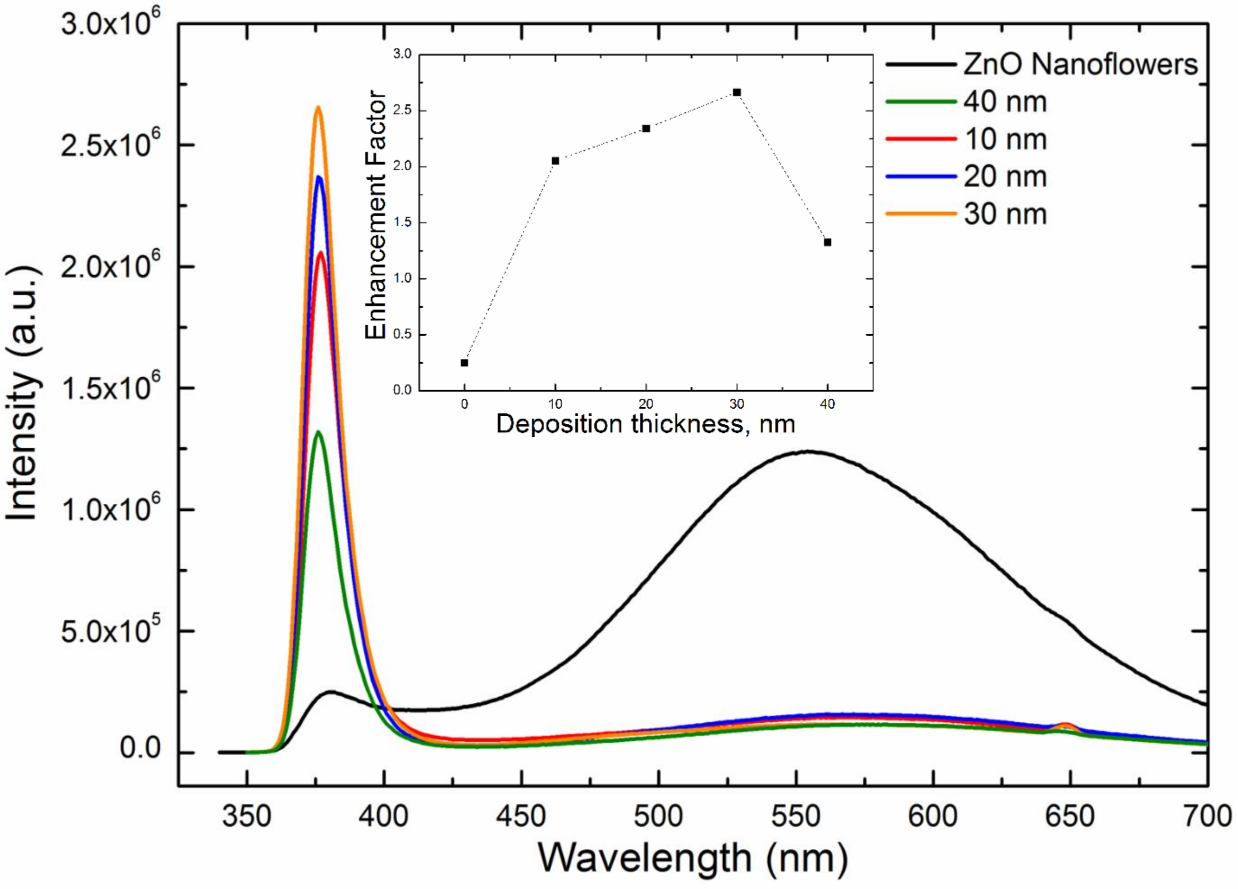

Supplementary Figure 2: The room temperature PL spectra of the ZnO nanoflower and spotted nanoflower with (i-iv) 10,20,30 and 40nm thickness. Insets show the corresponding variations of enhancement factors of UV with different deposition thickness of Au NPs (Enhancement factor is variation of UV emissions with different deposition thicknesses of AuNPs).

Supplementary Figure 3

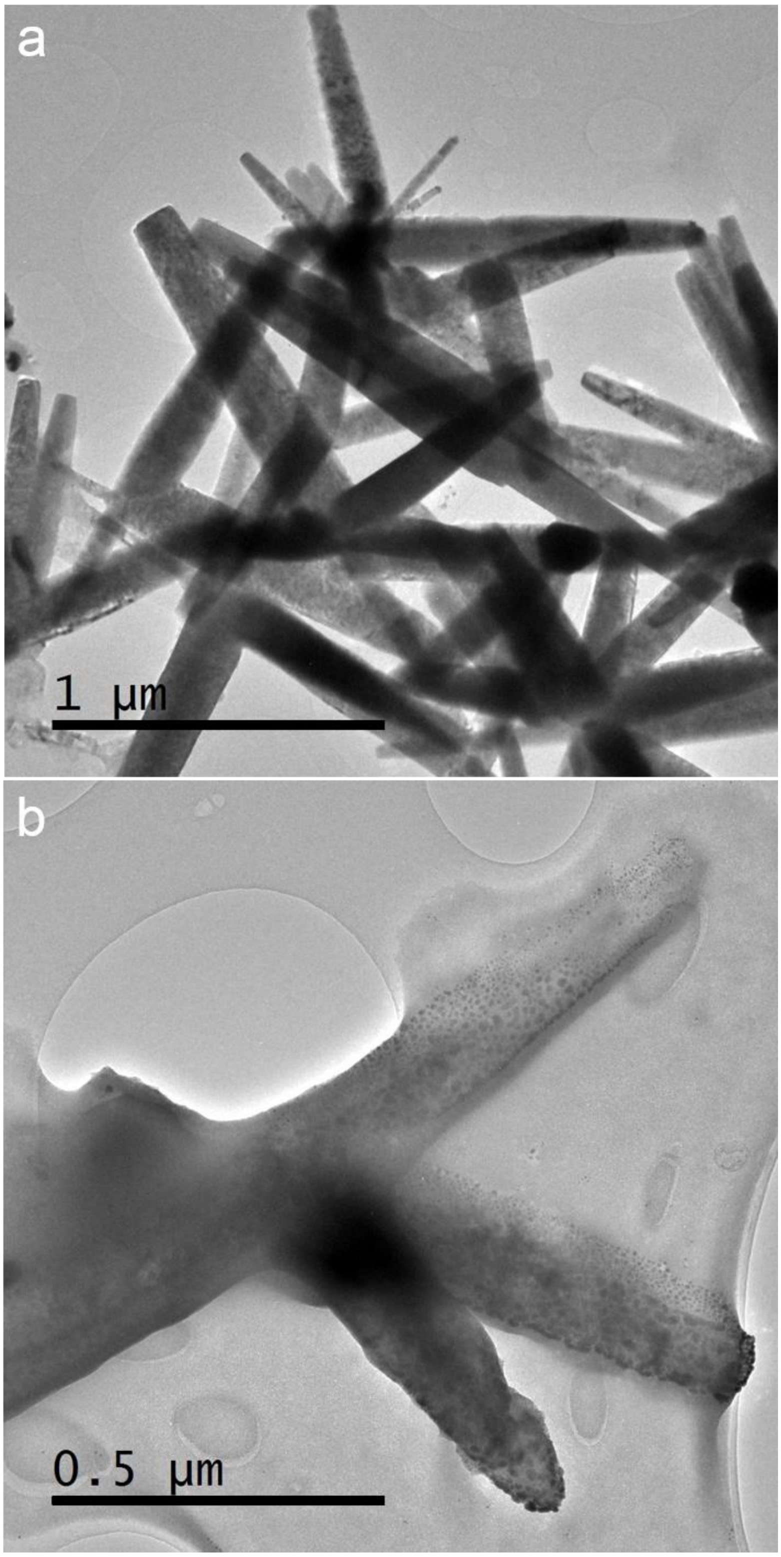

Supplementary Figure 3: TEM images of spotted nanoflower depicting the length.

Supplementary Figure 4

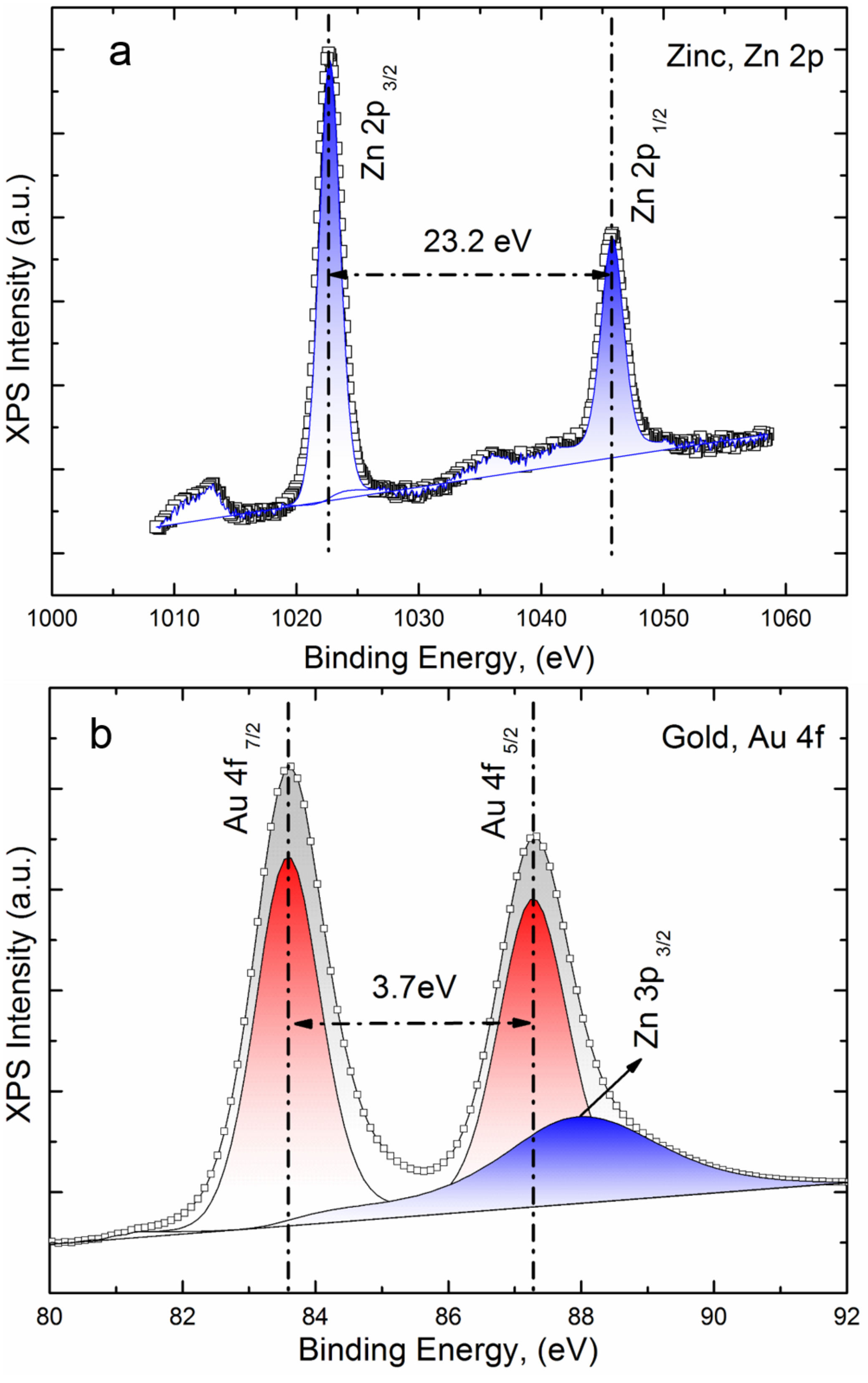

Supplementary Figure 4: The XPS spectra for a) Zinc, Zn 2p and b) gold, Au 4f obtained from spotted nanoflower.

Supplementary Figure 5

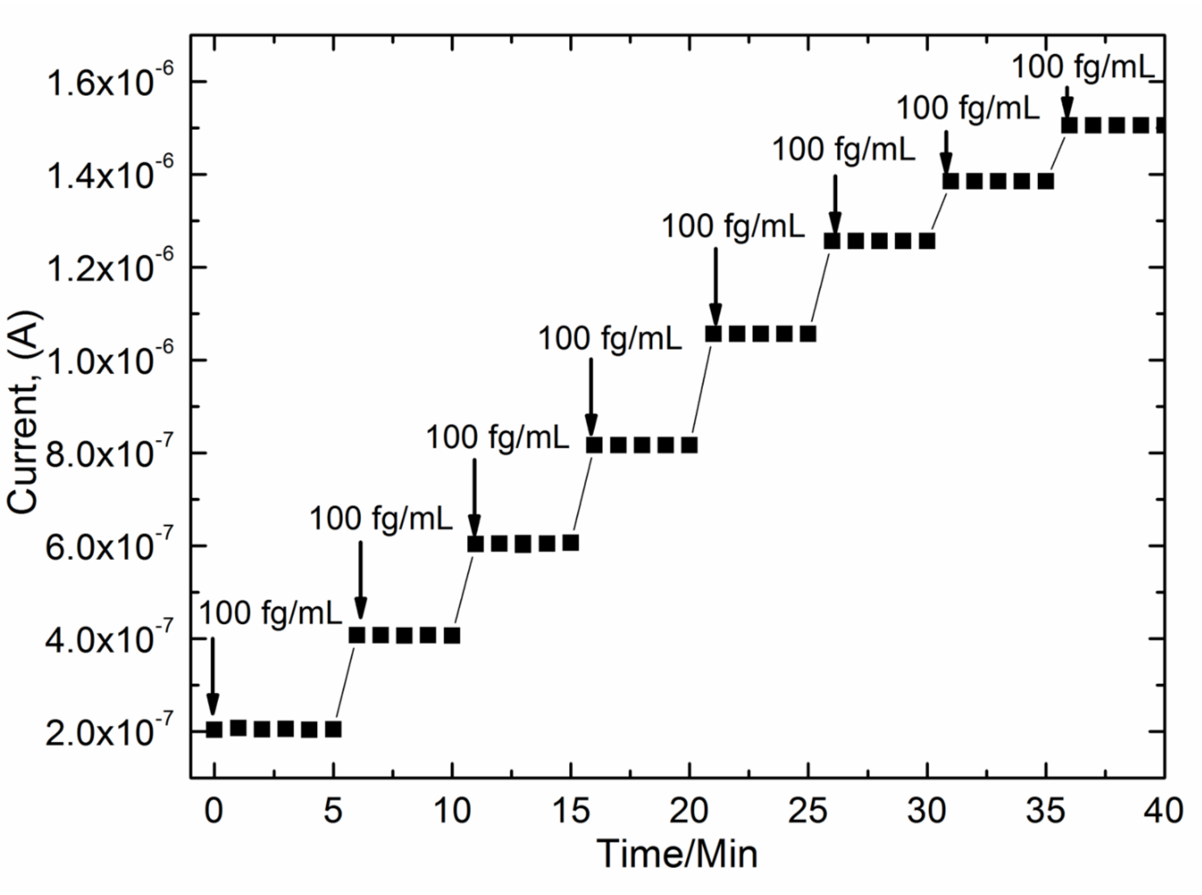

Supplementary Figure 5: The electroanalytical curve for 100fM concentration with 1 min response time.

Supplementary Table 1

S. Table 1: Binding energies of spotted nanoflower shows in figure 4 and chemical assignment.

Core level	Binding energy	Chemical moiety	Initial ZnO/Au (a)	Immo on ZnO/Au (b)	Hyb on ZnO/Au (c)
**Carbon, C1s**	284.7-286.2	C–C, C=C	284.72	284.47	284.79
	286-287	C–N, C–NH2, N–C–N, N–C=N	286.37	286.75	286.35
	287-289	N–(CO)–N, N–C=O, N–C(=O)–N	289.09	288.97	288.64
**Phosphorus, P2p**	133-134	$2p_{3/2}$	-	133.55	133.40
	134-134.9	$2p_{1/2}$, PO_4^-	-	134.10	134.05
**Oxygen, O1s**	531	C=O	531.23	531.10	531.05
	532-534	C-O-C, PO_4^-	532.73	532.60	532.50
**Nitrogen, N1s**	399-401	C–NH_2, NH_3^+, C=N–C; N–C–O, N–C=O,	-	399.80	399.40

Supplementary Table 2

S. Table 2: Modelled RC parameter for spotted nanoflower/p-DNA hybridized with different complementary DNA concentration.

DNA concentration (ng/mL)	Rct (MΩ)	CPE(nF)	Surface Concentration (@)	n value
**1^-6**	10.41	162.70	0.95	0.93
**1^-7**	9.78	136.30	0.95	0.93
**1^-8**	8.79	106.80	0.95	0.93
**1^-9**	6.68	92.90	0.94	0.93
**1^-10**	4.45	71.97	0.90	0.94
**1^-11**	2.81	57.10	0.85	0.95
**1^-12**	1.89	45.33	0.77	0.96
**1^-13**	1.01	35.58	0.57	0.97
